# ‘I heard about this study on the radio’: using community radio to strengthen Good Participatory Practice in HIV prevention trials

**DOI:** 10.1186/1471-2458-14-876

**Published:** 2014-08-26

**Authors:** Bonnie-Jeanne Medeossi, Jonathan Stadler, Sinead Delany-Moretlwe

**Affiliations:** Wits Reproductive Health and HIV Institute, Faculty of Health Sciences, University of the Witwatersrand, PO Box 18512, Johannesburg, South Africa

**Keywords:** Good participatory practice, Community radio, Clinical trials, HIV prevention, South Africa

## Abstract

**Background:**

During the Microbicides Development Program (MDP) 301, a clinical trial of a candidate microbicide amongst women in Johannesburg, South Africa, we used community radio to promote awareness of the trial, to inform community members about specific medical research procedures and terminologies, and to stimulate dialogue between researchers and local citizens.

**Methods:**

We used mixed methods to undertake a retrospective analysis of the social responses to the radio shows, focusing specifically on recruitment and participation in the MDP301 trial. We collected quantitative data that describes the themes and listener responses, the costs per broadcast, and the impact of the radio broadcasts on trial recruitment. Qualitative data on local reactions to the shows was gleaned from in-depth interviews with trial participants.

**Results:**

Over a seven-year period, 205 individual broadcasts were made on two separate community radio stations. Show themes were either specifically related to medical research issues (36%), or focused on general health issues (46%), and sexual and reproductive health, including HIV prevention (18%). 403 listeners made telephone calls to the radio station, and 12% of women enrolled as participants in MDP301 (n = 9, 385) reported that they had first heard about the trial from the radio. Qualitative interviews (n = 401) with female MDP301 participants highlighted the effects of the radio shows in making women aware of the trial, impressing them with the importance of health screening and knowledge, legitimizing trial participation, and stimulating dialogue between trial participants and their male partners.

**Conclusions:**

Community radio is a potent tool for raising awareness and local knowledge about medical research and, in addition to other methodologies, can be used to promote recruitment into clinical trials. We suggest that future HIV prevention trials consider an investment in community radio beyond recruitment advertisements that incorporates this into the broader community engagement plan as a key element of Good Participatory Practice in clinical trial settings.

## Background

For the past two decades, several trials of innovative biomedical HIV prevention technologies, such as intra-vaginal microbicide gels, have been undertaken. These studies involve thousands of participants drawn from communities that may or may not be familiar with medical research [1]. Limited understanding of research combined with inadequate communication between researchers and involved communities, as well as a lack of community involvement can result in misunderstandings and bioethical disputes, leading to the premature closure of trials [2]. Recognising the need to improve community – researcher communication and collaborations, UNAIDS and AVAC published the 1^st^ (2007) and 2^nd^ (2011) editions of the *Good participatory practice (GPP) guidelines for biomedical HIV prevention trials* [3]. The guidelines stress the need for effective community engagement throughout the life of an HIV prevention trial, and describe optimal practices to follow when designing and implementing research. Recommendations include multiple stakeholder advisory mechanisms and a stakeholder engagement and education plan. The guidelines also recommend that ‘effective stakeholder education is key to building research literacy, and ultimately, empowering community stakeholders as decision-making agents’ [3]. By building research literacy and educating stakeholders about HIV and HIV prevention, it is anticipated that the relationships between researchers and trial participants may become more equitable.

The GPP guidelines identify both formal and informal stakeholder advisory mechanisms, including community advisory boards (CAB), public discussion groups and community radio, which should be used to ensure continuous dialogue about clinical research and specific trials. While researchers often use the CAB structure as the primary mechanism for promoting community engagement in trials, this alone may often be insufficient to build local understandings of the research process. Limited understanding of research by CAB may also make it difficult for members to effectively communicate complex research concepts [4]. Individual members may also be insufficiently networked with all sectors of the community, despite the requirements for them to be representatives. Given the potential limitations of the CAB to facilitate effective communication between the researchers and trial communities, other forms of communication and community engagement mechanisms are needed.

Community radio is recommended by the GPP guidelines as ‘an informal’ engagement mechanism. As numerous commentators have noted, radio is a critical medium in reaching marginalised communities [5]. Radio is a powerful medium for communication with almost universal coverage [6]. Public health campaigns globally use radio for communication of health promotion messages, and some have had considerable success in improving knowledge around sexual and reproductive health [7–9]. To a lesser extent, radio has been used as a recruitment method for HIV prevention trials in different settings [10–12], although the outcomes have not been well documented. Studies in the US have reported on the cost benefit of using multiple media (e.g.: television, radio and newspaper) per participant recruited, yet little or no work has been done on the effectiveness of community radio for recruitment into trials in low and middle-income countries [12].

In South Africa, over 76% of households have access to a radio. Community radio stations were first established in South Africa following new legislation (The Independent Broadcasting Authority Act of 1993) governing the issuing of broadcast licenses. More than 100 community radio stations have registered since then, although many are struggling [13]. All registered stations in South Africa are required to include programs that have a social benefit to the listening community, such as educational, and health promotion shows.

In this paper we describe a weekly community radio show, *Tshireletso Health Talk* (THT), initiated by the Wits Reproductive Health and HIV Institute (Wits RHI), a South African research institute, as an additional and complementary community engagement mechanism for HIV prevention trials. Our aim is to assess how the radio broadcasts contributed toward community engagement, through an analysis of the show’s content, responses from listeners, and to explore whether this led to actual participation in the trial itself. In addition to these key issues, we briefly examine the costing implications for these activities in clinical trials.

We draw our data largely from the Johannesburg study site of the Microbicide Development Program (MDP) 301 trial. MDP301 was a multi-centre, randomized, double blind, placebo controlled trial that aimed to determine the efficacy and safety of PRO-2000/5 gel in preventing vaginally acquired HIV infection. PRO 2000/5 is a polymer gel applied vaginally using a disposable applicator. The gel was investigated to assess its ability to block the entry of sexually transmitted disease, including HIV pathogens into human cells. Women (n = 9, 385) were randomized to one of three gel arms: 2% PRO-2000/5, 0.5% PRO-2000/5, and a placebo gel as a control arm. In 2010, the trial reported that PRO-2000/5 gel was safe to use but ineffective in preventing HIV transmission [14].

## Methods

### Design

This is a post hoc analysis of data drawn from four sources: (1) audio recordings of radio broadcasts from 2003–2010 (n = 207); (2) MDP 301 participant files (n = 1708); and (3) qualitative in-depth interviews (n = 401) with 150 female participants in MDP 301; (4) project budget reports.

### Data collection and analysis

1. Audio-recordings of the radio shows were reviewed for the period 2003 to 2010. These were assessed for length, program topics, and listeners telephone calls and short message service (SMS). Researchers listened to the recordings of each show and prepared summaries of the content and categorized these according to the emergent themes. This data was entered into Microsoft Excel. Statistics were used to describe the number of shows broadcast during the time frame, proportion of these shows under different themes and the number of MDP301 trial participants who had heard about the study through the radio.
2. In order to further assess the radio show as a recruitment aid, we analysed the MDP301 participant locator files that contained information on where participants had head about the trial (‘Where did you hear about the trial?’). Participants were allowed to choose multiple options and possible options included: ‘another participant,’ ‘local clinic,’ ‘friend or family,’ ‘radio,’ and ‘study staff.’ For the purpose of this study, 1708 locator files were reviewed and summarised using Microsoft Excel.
3. To understand how the radio broadcasts may have helped individual women to make decisions about participating in the trial, qualitative interviews with MDP301 participants were also used in this analysis. For the social science component of the MDP301, a subset of women enrolled in the trial was randomly selected to participate in in-depth interviews (IDI) at weeks 4, 24 and 52 after trial enrolment. The interviews were conducted in the home language of the participant (SeSotho or IsiZulu), usually by an interviewer of the same sex, in a private interview room in the trial clinic. During the interviews, participants were asked where they had heard of the MDP trial and why they had decided to volunteer. A detailed description of the methods and results is documented elsewhere [15]. IDI were recorded on digital recorders, transcribed and translated into English and incorporated into a database in Nvivo2 (QSR International). Transcripts were coded in Nvivo using descriptive nodes developed by the MDP social science research team. These focussed on gel use and acceptability, condom use, sexual partnerships, trial experiences, and vaginal cleansing. Transcripts were coded by site staff and checked for consistency by a central coordinator who reviewed site data [15]. Additional codes were developed at site level *in vivo* to address specific needs using Grounded Theory [16]. For the present analysis, a word search for ‘radio’, ‘Jozi FM’ and ‘Thetha FM’ was used to identify texts that referred directly to the radio broadcasts. These texts were then analysed thematically.
4. Costing data was collated by reviewing the project specific budgets of clinical trials (2003 to 2010). The radio cost was typically included as a line item for community engagement activities in each study budget.

The Witwatersrand University Human Research Ethics Committee approved the MDP301 (Reference: 050108) and all participants provided written informed consent prior to enrollment.

Our research adheres to the standards contained in the Qualitative Research Review Guidelines (RATS) (http://www.biomedcentral.com/authors/rats).

### Description of the community radio stations

The *Tshireletso Health Talk* (THT) is a community radio show broadcast on two community radio stations in Johannesburg, South Africa: Jozi FM in Soweto and Thetha FM in Orange Farm. Jozi FM was established in 1999 and is currently the largest community radio station in South Africa. It caters to the Soweto with a population of 1.2 million inhabitants [17], and programmes cover local news, current affairs, sports, health matters, love, relationships and other family and social matters. Despite being a community radio station, Jozi FM has a commercial radio orientation. The Jozi FM presenters are all Black South Africans, and predominantly male; all are residents of Soweto [18].

Thetha FM was established in 1997, gained licensure in 2003, and started broadcasting to Orange Farm residents in 2005. Orange Farm is more rural in appearance than Soweto, and has a smaller population of 170 000 [19]. Thetha FM’s objectives are: to promote primary health care and community development; to empower the local community; and to motivate for positive social change [20]. Thetha FM broadcasts music and talk shows on variety of topics including health, education, current affairs and relationships. Programmes are specifically targeted at different population groups including women, children, youth and people with disabilities. Thetha FM presenters are all Black South Africans, and while they are residents of Orange Farm, many are migrants from all over South Africa, and have varying levels of education. Both radio stations have relatively small footprints, catering largely for communities in southwest Johannesburg. Jozi FM has a listenership of 564,000, while Thetha FM has 171,000 listeners [21].

### The Tshireletso Health Talk Show (THT)

The THT show commenced on Jozi FM (Soweto) in 2003 and on Thetha FM (Orange Farm) in 2005. Originally intended to raise awareness about specific trials, the shows also addressed general health topics, while maintaining a connection between the particular health topic and research issues pertinent to that topic. The show presenters had varying levels of education, ranging from community educators with a high school diploma through to professional nurses, doctors and academic staff, and some were residents of Orange Farm and Soweto. A typical show included a script with key messages that was vetted by senior research staff prior to being broadcast, a question posed to listeners and a scripted interview with a researcher, clinician or research nurse involved in the trials’ From mid-2009, the show scripts also included a fictional story or scenario that illustrated the social aspects of the issue under discussion. This modification stemmed from a reflection on the process by research staff, driven by feedback and suggestions from the radio station staff, and callers.

Since inception, the THT on both stations was open to listener call-ins throughout each show. Listeners were invited to call into the station at any point during the show. For each caller, the on-air expert responded to the comment or question. If they were unable to answer the question, the caller’s name and contact number was noted and a clinician or nurse followed-up on an individual basis within 24 hours. If necessary, callers were advised to seek treatment and care at their local primary health care clinic.

## Results

### Show content

Table 1 summarises the content of shows, categorised by whether they were specifically research-related, or focussed on general health education, or a combination of the two. Of the 205 shows reviewed, 36% of shows addressed issues related to research or trial-specific procedures. These shows provided information about planned, current, and completed trials in the community, as well as providing education about specific research terms or procedures such as ‘placebo’, ‘randomization’, and ‘double-blind’. A similar proportion of shows dealt with issues related to sexual and reproductive health (SRH) and HIV. Approximately 18% of shows dealt with issues relating to SRH, as this related to clinical trials. The emphasis in the shows was to promote understanding for the rationale for HIV prevention trials, for example by drawing attention to the high rates of HIV incidence amongst women and the need for female controlled methods of prevention. The remainder of shows (46%) provided more general health information.

**Table 1:** **Tshireletso Health Talk Radio Show programme topics, by category type (2003–2010)**

Topic	No of shows	Proportion of total shows (%)
Research-related	74	36
Health education	95	46
Combined research and health education	36	18
**Total**	**205**	**100**

### Listener responses

Table 2 summarises the total number and distribution of caller responses to the show in the period 2003–2010, categorised by whether they were related to research, general health education or other issues. For the total 205 shows on both stations combined, 403 phone calls were recorded, reflecting a mean of 1.9 calls per show (range 0–13). The shows that generated the most calls dealt with SRH issues such as sexually transmitted infections and the most popular presenters who attracted the majority of the calls were female and spoke a local language.

**Table 2:** **Tshireletso Health Talk Radio Show programme caller responses, by category type (2003–2010)**

Category	Total count n = 403	Proportion of total categories (%)
Research	73	18
Health and diagnostics	270	67
Other	60	15
Total	403	100

Eighteen per cent of calls dealt with specific questions about research. Callers were particularly interested in the microbicide gel that was being evaluated in the MDP301 trial and wanted to understand how the research products worked, and what the implications were for them if they participated in research. For example:

If I am gay am I allowed to use the [microbicide] gel to perform oral sex? (Female caller, Jozi FM; 4 October 2007).

Using the [microbicide] gel, how many rounds of intercourse does it last? (Female caller, Jozi FM; 4 October 2007).

Statistics say that women are the ones who mostly get HIV. Why are you doing HIV prevention in men? (Female caller, Thetha FM, 8 April 2009).

Many (67%) of the callers asked health-related questions, particularly about their sexual health. The following examples reflect the demand for specific information on genital symptoms and the diagnosis of sexually transmitted infections:

I am an 18-year-old girl and I have sores that bleed. Is it herpes? (Female caller, Jozi FM, 6 December 2007).

The skin around my vagina is itching; does it mean that I have a STI? (Female caller, Thetha FM; 30 August 2008).

If I sleep with a woman that is menstruating without a condom am I at risk of getting a STI? (Male caller, Jozi FM, 16 October 2007).

Callers also used the opportunity to seek advice on relationships, particularly how to talk about sexual health matters with partners, but also with children, as the following examples illustrate.

I have this problem that the guys I date, when I tell him ‘baby you are not satisfying me’ they run away or say I am cheating on them. So what can I do? (Female caller, Jozi FM; 27 July 2004).

How do I have to educate my child about sexual practice and condom use? (Female caller, Thetha FM; 12 November 2005).

### Awareness and dialogue about the MDP301 trial

Twelve per cent of women enrolled in the MDP301 trial reported joining as a direct result of hearing about the study on the radio (Table 3). For example, this trial participant describes how she was initially introduced to the trial through the radio show:

**Table 3:** **Participant-identified source of recruitment into the MDP301 Trial (2003–2010)**

Source	Total no. of participants	Proportion of participants (%)
Friend/Family	700	41.0
Another participant	417	24.4
Study staff	282	16.5
Radio	204	11.9
Clinic	101	5.9
No response	4	0.2
**Total**	**1708**	**100.0**

I heard from Jozi FM. I was at home when I was listening to the radio and I heard them talking about the study and they said there is a clinic called Tshireletso that is going to help people with HIV. Then I thought it is better that I join the study here at the clinic.

In addition to promoting trial recruitment, the radio show encouraged conversations about the trial in the community. While most participants heard about the MDP301 study from a friend or family member (41%), or ‘another participant’ (24.4%) (see Table 3), there is evidence that the radio shows stimulated local conversations that led women to volunteer to join the trial, as these quotes from interviews illustrate:

I heard about it on the radio and I kept telling myself that I would go. I wanted to join. My little sister at home is a nurse, and when I told her about what I heard, she told me to just go and join the study. She told me that it was going to help me but I never got around to doing it. Eventually, my little cousin sister came and she told me more about that study and also told me that I should join. Then eventually I went to the clinic and I ended up enrolling. I just wanted to get some knowledge about some of the things that I didn’t know about, instead of just sitting at home.

I heard about the study for the first time on the radio Jozi FM. And then I heard my friends talking about the study and they told me that there is a gel that is being used that they get from the clinic to protect themselves against HIV and STIs.

I heard about this study on the radio and did not pay much attention. I heard about the study again on the radio and told myself I will come and join but still I took my time. Then I met one girl whom I fellowship with. She told me about the study and I told her that I heard about it on the radio, I asked her if it was a serious thing she said it is fine because they even check everything about your health you will be satisfied. When I heard it on the radio again I decided to come.

As these excerpts show, listeners to the radio show sometimes misinterpreted the key messages being communicated. For instance, listeners interpreted the information they heard on the radio show as meaning that the gel was effective in preventing HIV, and not an experimental method being tested.

Listeners to the radio responded positively to broadcasts about sexual and personal health concerns. This prompted women to think about their health and seek further advice from the MDP clinic. A trial participant from Soweto recalled that she became concerned that she may have acquired an STI, and volunteered for the study for that reason.

I knew beforehand that I did not have HIV but I thought that maybe I had STIs that I did not know about. Like as they have said on the radio that some people might be having STIs without knowing it. That is what made me to be interested to come here.

Participants were also motivated to join the trial when they heard about particular investigations performed by the trial clinic that are not performed routinely within the public health sector, as well as by the desire to take responsibility for and control of their SRH, as these quotes illustrate:

What made me join the study was that I heard about it on radio, they were talking about different tests that they are performing and I got interested.

When I heard that they do HIV testing I told myself that I want know my health status. I heard about the study on the radio and I told myself that it is better I join it.

The radio show also reinforced trial specific requirements. For example, vaginal douching and using intra-vaginal products was a particular concern for the trial as this could interfere with the mechanism of the microbicide gel.

They said that it is not good to insert fingers in the vagina. Even on the radio they said that women shouldn’t insert fingers in the vagina [to clean]. If there is dirt [in the vagina] it will come out on its own.

Some female participants in the trial struggled with disclosing their participation and study gel use to their partners. The radio show made male partners more aware of the trial increasing their receptiveness to the idea of women’s participation. This participant from Soweto recalled how her partner changed his perception of the trial after hearing about it on the radio.

I think that before, he did not believe that this gel is something that is true and that everyone uses it, then as time went on he heard about the gel on the radio and then he agreed with it.

Moreover, the radio show facilitated conversations with sexual partners, as the following quotes illustrate:

I asked him after sex how did he feel and he said it was okay and then I told him I heard about the study on the radio and I went to Baragwanath to join it. They are researching the gel but we are told to use it together with a condom because they are still doing a research.

People are not the same; sometimes it is not easy for a woman to talk about certain things with a man. I have heard from the radio that we also have to involve our partners like if they want to see when we insert the gel they have to watch us inserting it.

### Costing

The radio show was aired on both stations on a total of 205 occasions: 163 on Jozi FM and 42 on Thetha FM. The mean length of one episode on Jozi FM was 30 minutes (range 10 to 50 minutes) while the mean length of an episode on Thetha FM was 54 minutes (range 20 to 60 minutes). The overall cost of the radio show over the seven-year period was ZAR 793 694 (USD 73 138, 03), based on current exchange rates (USD 0.09 = ZAR 1), with a mean cost of ZAR 3 834 (USD 353,30) per show. Contract costs increased incrementally on an annual basis.

## Discussion

As biomedical research expands globally into settings that have little or no experience of clinical trials, so the need for improved communication increases. Divergent understandings of research, ethics, consent, evidence, and biomedical procedures requires dialogue between researcher and community [22]. Recognising these challenges, biomedical researchers have drawn on participatory methodologies to promote better local understandings about clinical trial procedures [23] and involved community structures in the management of trials (CAB) [24]. Community radio is, as we have suggested, an integral aspect of the overall strategy of community engagement. Beyond providing a tool for recruitment into the trial, the radio show described here served as a stimulus for local dialogue about general health issues (primarily SRH and HIV), which in turn encouraged women to visit the research clinic to learn more about their health. This ultimately promoted trial recruitment and enrolment into the MDP301 clinical trial. The program also supported MDP 301 trial participants’ disclosure of their participation and gel use with sexual partners.

In other similar settings it has been shown that trial participation depends largely on people having a thorough understanding of the clinical research process [25]. By investing in public health education programs which promote awareness and engagement in trials, the general public may become more willing and able to participate knowledgeably in research [25]. Similarly, our assessment of community radio to support the MDP301 trial suggests that radio was an important information source about biomedical research.

The interactive format of the show that encouraged listeners to call in and exchange their views with the presenters was critical for the radio show’s success. It provided a forum for information seeking about personal health issues, which in turn created opportunities for the researchers to raise awareness about HIV prevention research. By posing questions and presenting the fundamentals of clinical research, the radio show began to stimulate a dialogue between researchers and local community members, as well as within the community. Women spoke to their friends and family about their decisions to enrol in the trial based on what they had heard on the radio. This also demonstrates the power of social networks and interpersonal communication to influence women’s decisions to learn more about their health and to participate in clinical trial research [26].

GPP is a necessary component of clinical trial research, particularly in research-naïve and vulnerable communities, in order build trust and increase research literacy. Community radio is an important engagement mechanism in low and middle-income settings where most households have access to a radio. However, radio alone is not sufficient for research literacy or to promote local level dialogue about clinical trial research. This may only be possible by using a mix of mass media and interpersonal communication, such as SMS and organised community dialogue sessions.

South Africa possibly presents a unique case for the use of community radio in the promotion of clinical trial research, due to its well-developed and autonomous community radio programing policy that supports community engagement in local developmental issues. Although similar communication initiatives exist in other African settings [27], that broadcast health education and promotion, particularly on issues relating to reproductive health and HIV/AIDS [8], the program reported on in this paper is one of the first initiatives to promote engagement with clinical trial research. Our experience of community radio for public engagement in medical research can serve as a model for similar developments elsewhere.

While our analysis shows that radio is a useful community engagement mechanism, we acknowledge certain limitations. The radio program described here focused on recruiting female participants into a clinical trial that targeted women. However there is some evidence in our data that suggests that men listened to the show and that this may have encouraged male partners to support women’s trial involvement by legitimizing the trial.

Another limitation is that the call-in format excluded listeners without access to phones or sufficient funds to cover the cost of a call. Therefore we presume that our data excludes many of those who were unable or did not want to spend money on calling into the talk show. However, a SMS and call back system catered for those who required more lengthy consultations. Moreover, considering that there are eleven official languages in South Africa and the communities in this research include diverse populations, there may have been a language barrier for some listeners if the presenter was not speaking in their first language.

## Conclusion

There is growing recognition of the need to build greater participation of communities and beneficiaries in the development of HIV prevention research trials. The GPP guidelines recommend the use of community radio, although there is little empirical evidence to support these recommendations. Our research provides preliminary evidence that a regular community radio show with an interactive format can promote a continuous community dialogue between researchers and the community, and enhanced trial participation. It is anticipated that these findings may be explored in different settings to further develop radio as part of the GPP toolkit.

## Abbreviations

CAB: Community Advisory Board
GPP: Good participatory practice
HIV: Human immunodeficiency virus
IDI: In Depth Interview
MDP: Microbicides Development Program
THT: Tshireletso health talk
SMS: Short message service
SRH: Sexual and reproductive health
USD: United States Dollar
Wits RHI: Wits Reproductive Health and HIV Institute
ZAR: South African Rand.
